# Manufacturing of 3D-Printed Microfluidic Devices for the Synthesis of Drug-Loaded Liposomal Formulations

**DOI:** 10.3390/ijms22158064

**Published:** 2021-07-28

**Authors:** Giulia Ballacchino, Edward Weaver, Essyrose Mathew, Rossella Dorati, Ida Genta, Bice Conti, Dimitrios A. Lamprou

**Affiliations:** 1 School of Pharmacy, Queen’s University Belfast, Belfast BT9 7BL, UK; giulia.ballacchino01@universitadipavia.it (G.B.); eweaver01@qub.ac.uk (E.W.); emathew01@qub.ac.uk (E.M.); 2 Department of Drug Sciences, University of Pavia, Viale Taramelli 12, 27100 Pavia, Italy; rossella.dorati@unipv.it (R.D.); ida.genta@unipv.it (I.G.)

**Keywords:** microfluidics, chip manufacturing, 3D printing, nanoparticles, liposomes, curcumin, drug delivery, personalised medicine

## Abstract

Microfluidic technique has emerged as a promising tool for the production of stable and monodispersed nanoparticles (NPs). In particular, this work focuses on liposome production by microfluidics and on factors involved in determining liposome characteristics. Traditional fabrication techniques for microfluidic devices suffer from several disadvantages, such as multistep processing and expensive facilities. Three-dimensional printing (3DP) has been revolutionary for microfluidic device production, boasting facile and low-cost fabrication. In this study, microfluidic devices with innovative micromixing patterns were developed using fused deposition modelling (FDM) and liquid crystal display (LCD) printers. To date, this work is the first to study liposome production using LCD-printed microfluidic devices. The current study deals with 1,2-dimyristoyl-sn-glycero-3-phosphocholine (DMPC) liposomes with cholesterol (2:1) prepared using commercial and 3D-printed microfluidic devices. We evaluated the effect of microfluidic parameters, chip manufacturing, material, and channel design on liposomal formulation by analysing the size, PDI, and ζ-potential. Curcumin exhibits potent anticancer activity and it has been reported that curcumin-loaded liposomes formulated by microfluidics show enhanced encapsulation efficiency when compared with other reported systems. In this work, curcumal liposomes were produced using the developed microfluidic devices and particle sizing, ζ-potential, encapsulation efficiency, and in vitro release studies were performed at 37 °C.

## 1. Introduction

Over the past two decades, nanotechnologies have progressed steadily, resulting in structures, devices, and systems with innovative properties and functions. The application of nanotechnologies to human health led to promising results in branches of medicine concerning cancer and neurological, cardiovascular, and infectious diseases, with positive results also for the development of antiviral vaccines [1,2]. Moreover, several promising small molecule drugs and genes with issues of stability, solubility, and nonspecific toxicity can now be delivered using nanocarriers such as micelles, polymeric or liposomal formulations, and nanoemulsions [3,4]. Nanoemulsions have been widely studied for therapy and imaging and have the potential to be used as theragnostic agents for treating and monitoring tumours [4,5]. Among the various lipid-based formulations, liposomes have been extensively studied due to their full biocompatibility, ability to transport and protect encapsulated substances, improve their solubility, and provide a controlled release [6]. Liposomes are spherical vesicles with particle sizes ranging from 30 nm to several micrometres, consisting of one or more phospholipidic bilayers, which self-enclose in aqueous solution [7]. Among drug delivery systems, liposomes represent an advanced technology for the delivery of active molecules to the site of action, and can transport either aqueous or lipid drugs, depending on the nature of the drug molecule [7]. In addition, encapsulated drugs are more bioavailable and are protected from premature degradation and nonspecific side effects, therefore their toxicity is lower [7]. Thanks to these advantages, liposomes are promising systems for drug delivery applications. 

Liposome properties vary substantially with lipid composition, size, surface charge, and the method of preparation [8]. Bilayer stability was reported to improve following the addition of cholesterol, and the concentration of this fatty substance influences the encapsulation capacity and drug release [9]. Moreover, research conducted on the effects of cholesterol revealed a concentration-dependent relationship between the addition of cholesterol and an increase in vesicle size [10]. Vesicle size affects the circulation half-life of liposomes, and both size and number of bilayers have an influence on the amount of drug encapsulated in the liposome [7].

One of the conventional methods for producing liposomes is thin-film hydration, which is simple and widely applicable; however, limitations such as low encapsulation efficiency, process control, and scalability must be considered [11]. High encapsulation efficiency is achieved using the reverse-phase evaporation method; however, drawbacks of encapsulating the compound with an organic solvent include reduced stability of fragile drugs and the need for sonification [7]. Vesicle size reduction can be obtained also by extrusion, passing the liposome suspension through a membrane of defined pore size. Extrusion is the most efficient nanosizing technique given that it is simple and fast, with limitations in scaling-up [12]. Overall, the translation from the laboratory to large-scale production has been one of the major challenges for the success of nanoparticle manufacturing.

More recently, development of microfluidic techniques has addressed the scalability problem and led to the formulation of liposomes within a confined microenvironment [13]. Microfluidics allows precise control and manipulation of small volumes of fluids in a network of microchannels in contrast to the turbulent flow that occurs at the macroscale, since microfluidic mixing is not governed by the same laws as mixing at the macroscale [14]. The main feature of microfluidic techniques is the laminar flow in microchannels that cannot be achieved in macroscale devices due to a drastically different surface-to-volume ratio and reduced inertial forces at the microscale [14]. Microfluidic techniques offer advantages compared with traditional methods in terms of control of nanomaterial characteristics, process reproducibility, fast mixing, and high throughput [3,14]. Rapid liposome screening is achieved, thanks to the effectiveness and easy scalability of the microfluidic technique [15]. In addition, the parallelization, automation, continuous flow, and fast mixing used in industrial scale production increase the total output and reduce batch variability and human error, leading to less expensive nanomedicines [16]. Moreover, miniaturization of the fluidic environment leads to efficient use of materials and excipients [17]. This ability to reduce solvent use is likely to provide an economical and environmentally friendly technology [3].

**Figure 1:**
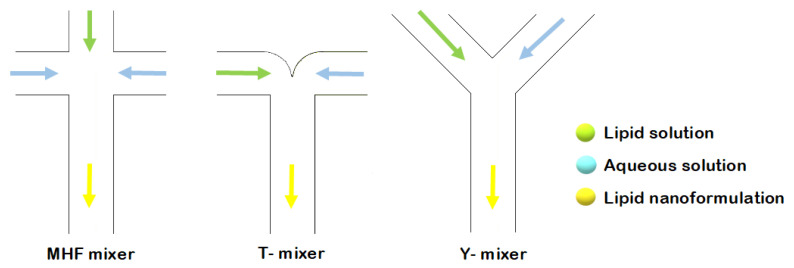
Schematic representation of liposome formation by MHF, and through T- and Y-shaped mixers.

In the application of microfluidics for liposome synthesis in lab-on-a-chip-based devices, various micromixers have been designed [18]. Altering channel geometry enables an increase in the contact area and contact time between the reagents involved in the mixing process. The different channel layouts include microfluidic hydrodynamic focusing (MHF), and T- or Y-shaped mixers (Figure 1) [18].

**Figure 2:**
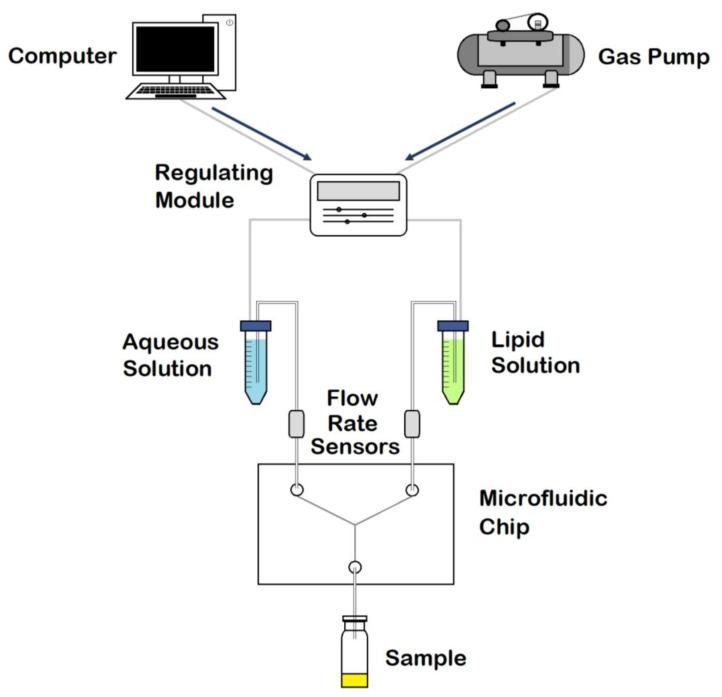
Schematic representation of the components in a microfluidic system. Adapted with permission from Weaver et al. [19].

In the current study, several microfluidic architectures were designed, consisting of Y-shaped mixers with different patterns, composed of two inlets and one outlet. A stream of lipid in alcohol solution and a stream of an aqueous solution flow through the two different channels. Liposome fabrication is achieved through the intersection of the two channels, where the two streams combine. Thus, mixing depends on the diffusion process at the interface between the two liquids, causing the precipitation of the lipids in aqueous solution to form micelles first, and liposomes after [11]. The spontaneous self-assembly process of liposomes is achieved at buffer concentrations above the micellar critical concentration. Above this concentration, the surfactant forms spherical vesicles having at least one lipid bilayer. The physical and chemical features of liposomes formulated by microfluidics can be modified by controlling the channel length, solution flow rates, composition, and environmental factors such as pH and temperature [17]. In conclusion, liposomal formulations produced using microfluidic chips offer advantages compared with conventional methods, thanks to the properties and dynamics of fluid flow. A scheme of the different components in a basic microfluidic system is shown in Figure 2. 

**Figure 3:**
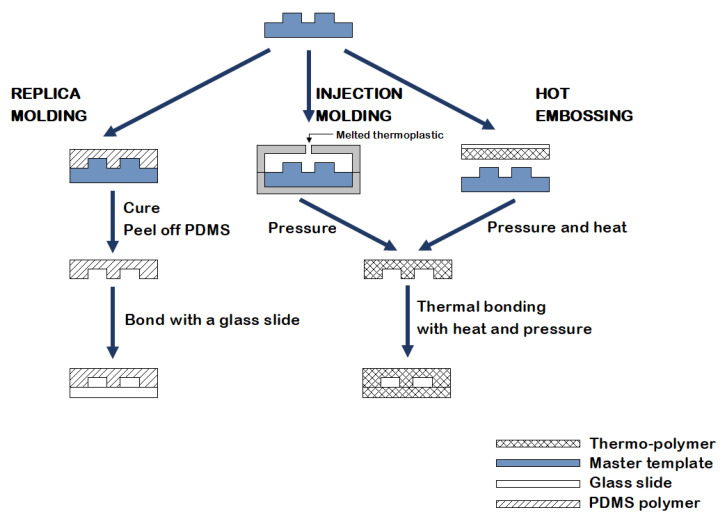
An illustration of the fabrication methods currently used in microfluidics: replica molding, injection molding, and hot embossing.

Several methods for producing disposable microfluidic devices have been reported in the literature [20]. Fabrication based on molding (Figure 3) includes replica molding, injection molding, and hot embossing [20]. Replica molding is a form of soft lithography that uses silicon molds with patterned features coated with a photoresist, over which liquid polydimethylsiloxane (PDMS) is poured and cured [17,18]. Sealing or bonding the mold with a polymeric or glass slide allows the creation of multilayer 3-dimensional microfluidic systems [21]. Due to the low cost, fast processing, reusability of the coated masters, and the possibility to create complex three-dimensional systems, this technology became a standard in the fabrication of microfluidic devices [21]. Furthermore, PDMS is a biocompatible polymer suitable for biological and cellular applications; however, the low compatibility of PDMS with organic solvents limits its use to aqueous solutions [21]. Both injection molding and hot embossing generate high-throughput, inexpensive, and precise microfluidics [20]. Injection molding consists of injecting a melted thermoplastic into a heated mold cavity under high pressure at a specific rate and then cooling it below the material glass transition temperature (Tg) before releasing it from the mold [20,21]. Drawbacks include the material restriction to thermoplastics and the high mold fabrication costs [20]. Hot embossing is a technique wherein sheets of thermoplastic materials are patterned against a stamp using pressure and heat, with limitations including its time consuming nature and the high cost of mold fabrication [20,21]. 

In recent years, 3D printing (3DP) has emerged as a new technology for the fabrication of microfluidic chips, building three-dimensional (3D) objects layer-by-layer by converting a 3D digital model into a physical object [20]. Compared with other fabrication methods, 3DP requires less fabrication time, lower expenditure, and is suitable for multiple uses [22]. Effective approaches that have been used successfully include fused deposition modelling (FDM) and resin 3DP technologies such as stereolithography (SLA) [23]. FDM is the most widely used 3DP technology, utilizing a heated nozzle fed with a thermoplastic material which is extruded and merges onto the previous layer before cooling down to a solid state [20,24]. FDM technology is inexpensive and widely accessible, and while several applications in microfluidics exist, compression stress fractures and limited resolution for producing microfluidic channels ultimately make FDM an unsuitable method for microfluidic applications [20]. Resin 3DP includes three different technologies: SLA, digital light processing (DLP), and liquid crystal display (LCD) [25]. All these technologies build objects layer-by-layer by photopolymerization of a liquid resin contained in a tank and cured against a build platform [25]. SLA, invented in the 1980s, is an ideal method of obtaining objects with clear details due to a laser that induces the polymerization of a liquid photosensitive resin [24]. While SLA allows for higher resolution and accuracy when compared with FDM and has the wherewithal to be the ideal method of choice for microfluidics, there is still room for development and enhancement. Instead of a single-point laser, DLP uses a digital micromirror device (DMD) that cures an entire layer simultaneously [24]. Liquid crystal display-based 3D printers use LED lights to cure resin in a similar way to DLP 3D printers but, instead of projectors and mirrors, they use LEDs shining through an LCD panel, which acts as a mask blocking off light to the areas that should not be solidified [25]. By flashing an entire layer at once, both DLP and LCD share the advantage of a quicker building time than SLA, as this technique does not depend on the bidimensional size of the object [25]. The current study focuses on the potential advantages and drawbacks of FDM and LCD printers and on the comparison of these methods with readymade polymeric chips; to the best of our knowledge, this is the first time that such a comparison has been made. 

After successfully printing the microfluidic chips, they were used to formulate liposomal nanoparticles (NPs). Finally, a drug molecule (curcumin) was used for encapsulation, and drug release studies were performed. Curcumin is a polyphenol extracted from *Curcuma longa* with therapeutic effects demonstrated in inflammation, arthritis, metabolic and neurogenerative diseases, and several cancers [26]. It exhibits its anticancer activity by targeting different cell signalling pathways. However, undesirable side effects, poor bioavailability, and low aqueous solubility hinder its application for commercial use [26]. Liposomes have demonstrated potential as carriers in order to overcome the limitations of curcumin by encapsulating the drug into particles. To the best of our knowledge, only one study has been conducted on curcumin-loaded liposomes produced by microfluidics, and this study used commercial chips with different dimensions and formulation parameters [27].

The main aim of this preliminary research was to investigate the ability of FDM and LCD chips to formulate stable particles and to examine, in depth, the influence of factors such as channel geometry and flow rate on the achievement of controlled particle characteristics. Moreover, this study particularly focused on the formulation and application of liposomes for the delivery of curcumin.

## 2. Results

### 2.1. Chip Design and Printing

Three-dimensional printing is a low-cost technology for manufacturing microfluidic devices which requires the following four steps:

- Step 1: creating a 3D design of the master using a computer-aided design (CAD) tool (e.g., Tinkercad).
- Step 2: slicing the CAD design into standard tessellation language (STL) format.
- Step 3: processing the STL file with the software of the printer to convert the 3D files into models (e.g., Ultimaker Cura for FDM 3D printers, Z-SUITE for LCD 3D printers).
- Step 4: printing the design with the 3D printer [22].

**Figure 4:**
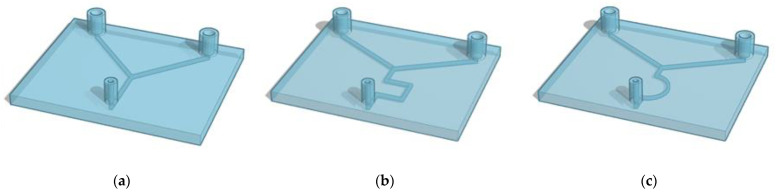
Representation of the 3D-printed microfluidic devices. (**a**) present 1 mm inlet channels that combine in one central channel that goes straight to the outlet point (Y shape). The same pattern of channels is designed both with a circular and squared (**a**) section. Devices (**b**) and (**c**) present 1 mm squared channels with zigzag- and half-moon-shaped channels, respectively.

The design of a potential device is key for structure and function of the final microfluidic device. The 3D-printed microfluidic designs studied in this work are shown in Figure 4. 

Microfluidic device fabrication with FDM-based Ultimaker 2+ using the NinjaFlex filament is a one-step process for flexible chips, which require less careful handling than those made from more brittle materials. Print settings were adjusted to reach a compromise between print quality and printing time. Lowering the infill density to 60% allowed for a faster printing time while ensuring controlled microscale fluid flow without leaking channels. Microfluidic devices fabricated with Zortrax Inkspire’s UV LCD technology had a faster printing time when compared with devices manufactured with FDM technology. Nevertheless, resin devices require a post-printing process with isopropyl alcohol to clean out the resin residues. 

All the printed devices underwent a syringe test with deionized water. The microfluidic channel test setup requires only a syringe pumping water into the two inlets; fluid flows through the channels and comes out of the outlet. The purpose of the test is to identify chip imperfections that can lead to channel occlusion or leakage from the surface of the chip.

Furthermore, the interaction of Zortrax Inkspire’s resin with the solvent used for the synthesis of NPs was studied. A resin sample was printed and immersed in ethanol. Over the first three days, the sample lost weight until it reached a plateau corresponding to a loss of 1%.

### 2.2. Nanoliposomal Formulations

**Figure 5:**
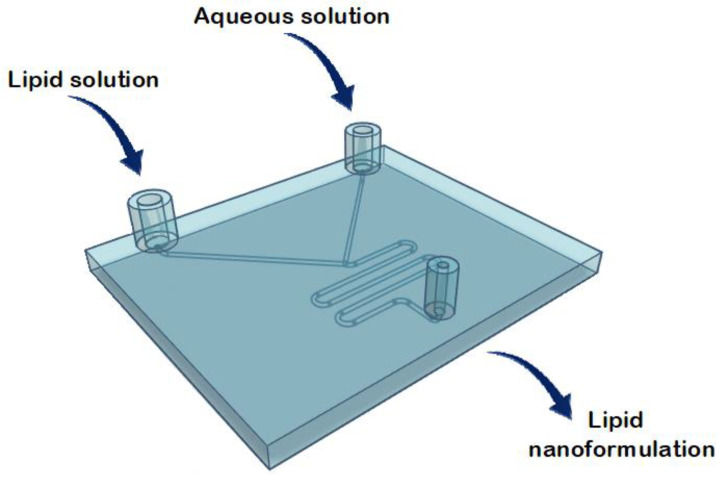
Representation of the structure of the commercial microfluidic chip for the lipid nanoformulations.

Liposomes were prepared using a commercial microfluidic device and compared with those prepared using 3D-printed microfluidic devices (Figure 4). The micromixers consisted of two stream inlets that merged into a microchannel (an example can be found in Figure 5).

Typically, a water-miscible organic solvent containing lipids and an aqueous solution are injected at the inlets [28]. Microfluidic mixing occurs at the intersection of the inlet channels when the two fluids combine, and lipids form a bilayer surrounding an aqueous core [28]. Therefore, the mixing process leads to liposome formation by the self-assembling mechanism [28].

A study by Hamano et al. examined how phosphocholine acyl chain length affects the resulting curcumal liposome formulations and, by comparing DMPC (C14), 1,2-dipalmitoyl-sn-glycero-3-phosphocholine (DPPC) (C16), and 1,2-distearoyl-sn-glycero-3-phosphocholine (DSPC) (C18), demonstrated that only DMPC formulations incorporate curcumin in nanoparticles with a small diameter and a narrow size distribution [27]. Moreover, longer acyl chains enhance molecular interactions and packing while the flexibility of DMPC formulations allows higher curcumin loading and liposomal stability due to increased curcumin–DMPC interactions [27]. Therefore, for the reasons mentioned above, DMPC was finally selected for use in the current study. Phospholipids (DMPC) were combined with cholesterol at a ratio of 2:1, as it was previously reported that a 2:1 lipid:cholesterol ratio gives the most stable liposome composition and controlled drug release of vesicles [29]. Lipids in combination with cholesterol were then dissolved in ethanol at a concentration of 1 mg/mL. The two chip inlets correspond to the lipid solution and the aqueous solution (PBS, pH 7.4), and batches were collected from the outlet port.

**Figure 6:**
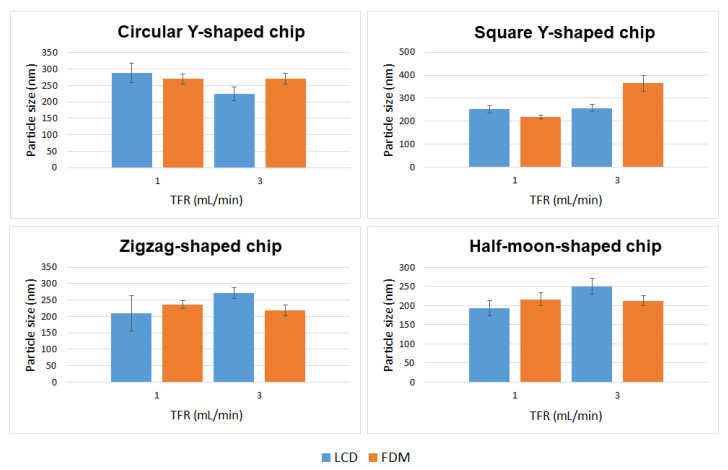
Mean particle size of DMPC liposome formulations prepared using circular Y-shaped, square Y-shaped, zigzag-shaped, and half-moon-shaped chips at TFRs of 1 and 3 mL min^−1^.

Applying different flow rate ratios (FRR) between the lipid and the aqueous phases, and different total flow rates (TFR, the sum of the flow rates of the two inlet solutions) allows for control of the particle size and stability of the liposomal formulation [19]. By varying the FRR of the solvent:aqueous phase from 1:1 to 1:3, the final solvent concentration is reduced, reducing the possibility of particle aggregation; however, since there is dilution before analysis, in this study changing the FRR did not significantly affect the vesicle size (data not included). An FRR of 1:1 (organic:aqueous phase) was therefore selected, and liposome features were investigated at TFRs of 1 and 3 mL min^−1^. Liposomes prepared at TFR of 1 mL min^−1^ using a commercial microfluidic device exhibited a size of 182.96 ± 23.56 nm, with a polydispersity index (PDI) of ~0.224 and ζ-potential and mobility measurements of −8.77 ± 3.48 mV and −0.68 ± 0.27 μmcm/Vs, respectively. Liposomes prepared at TFR of 3 mL min^−1^ exhibited a size of 231.29 ± 39.63 nm with a PDI of ~0.204, ζ-potential of −9.46 ± 2.04 mV, and mobility of −0.74 ± 0.16 μmcm/Vs. Liposomes were also prepared using circular Y-shaped, square Y-shaped, zigzag-shaped, and half-moon-shaped chips fabricated with FDM and LCD printers. Mean particle size, PDI, ζ-potential, and mobility were measured (Table 1) and results in terms of particle size and TFR are shown in Figure 6.

Liposome characteristics such as particle size and surface charge influence the blood circulation time and consequently also the passive targeting capacity of liposomes [30]. For example, 450–500 nm liposomes have higher reticuloendothelial system-mediated blood clearance rates than smaller ones; moreover, extremely small particles (less than 5.5 nm) are easily eliminated through kidney filtration [30]. Furthermore, in vivo studies demonstrated that the surface charge of liposomes influences cellular uptake and blood distribution [31].

Curcumin-loaded liposomes were also prepared using circular Y-shaped, square Y-shaped, zigzag-shaped, and half-moon-shaped chips fabricated by LCD and FDM printers. Liposomal curcumin was prepared by dissolving curcumin in the lipid phase due to its poor solubility in water. Five milligrams of DMPC, cholesterol (2.5 mg), and curcumin (1 mg) were dissolved in 1 mL of ethanol. The organic phase was then injected into the micromixer to mix with the aqueous phase. A flow rate ratio of 1:1 (organic:aqueous phase) was selected and liposome features were investigated at TFRs of 1 and 3 mL min^−1^. In drug-loaded liposomes, curcumin is solubilized in the phospholipid bilayer of the liposomes since the lipid domain provides a suitable hydrophobic environment. Curcumin was found to preferentially locate between the lipid chains in DMPC liposomes in previous fluorescence quenching studies, which proved that the preferable position for curcumin is along the entire length of the acyl chains as the size of the DMPC tails and the curcumin molecule are quite similar [32].

**Figure 7:**
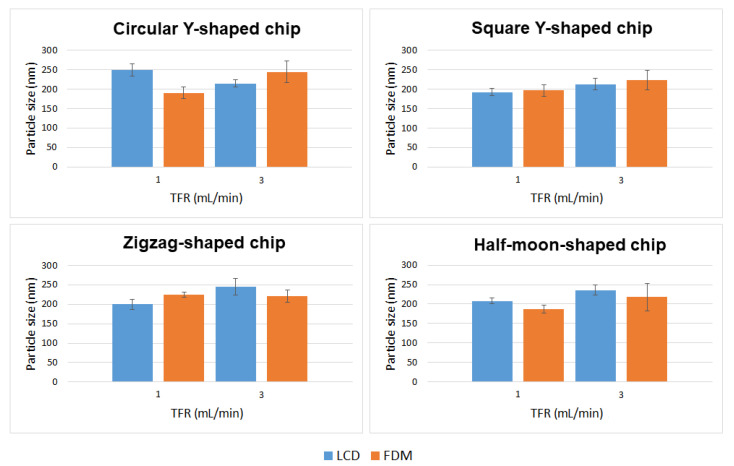
Mean particle size of curcumin-loaded liposomes prepared using circular Y-shaped, square Y-shaped, zigzag-shaped, and half-moon-shaped chips at TFRs of 1 and 3 mL min^−1^.

Mean particle size, PDI, ζ-potential, and mobility are shown in Table 2, and a comparison of liposomes in terms of particle size and TFR is shown in Figure 7.

### 2.3. Encapsulation Efficiency and In Vitro Release Studies

**Figure 8:**
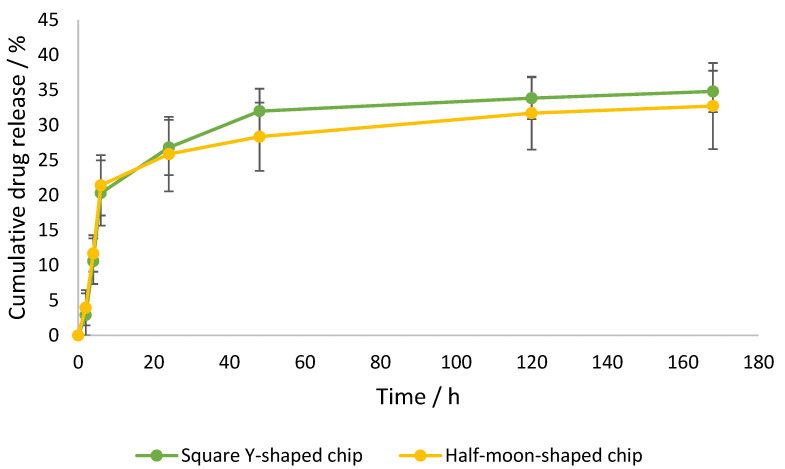
Cumulative curcumin release over time from liposomes formulated by square Y-shaped and half-moon-shaped chips, with a maximum release of 35%.

Dynamic dialysis is the first method of choice for the determination of release kinetics from NPs; therefore, drug release in the current study was investigated using this method [33]. Drug release in the receiver compartment requires two steps: first, release of the drug from the liposomes into the dialysis chamber; and second, diffusion through the dialysis membrane [33]. Curcumin-loaded liposomes were prepared using square Y-shaped and half-moon-shaped chips fabricated by LCD printer. To the best of our knowledge, this paper is the first to study liposome production using LCD-printed microfluidic devices. As previously reported in the literature, both the size and PDI of the resulting formulations decreased when the ratio of the organic:aqueous phase was decreased from 1:1 to 1:3; therefore, an FRR of 1:3 (organic:aqueous phase) was selected in order to achieve smaller particles, while the TFR was set at 1 mL min^−1^ [27]. This trend is due to the enhanced levels of hydrophobic interactions between the organic and aqueous phases, causing an entropic tendency for liposomes to form. Three repeats were conducted for each liposome suspension. When the curcumin concentration in the dialysis bag outer compartment was analysed after 1 h of incubation using a UV-vis spectrophotometer, no peak was detected, indicating that the encapsulation efficiency was approximately 99.9%. Since the concentration of encapsulated curcumin was impressively high after 1 h, nanocarrier reversible bonds with curcumin were hypothesized. It has been reported that there is direct competition between curcumin and cholesterol for lipid binding, however, cholesterol interaction with DMPC acyl chains is stronger than curcumin–DMPC interaction. Hence, cholesterol–DMPC interaction increases over time as the most stable product is formed at thermodynamic control [34]. Release profiles of liposomal curcumin in Figure 8 show an initial burst of drug release from both formulations. This significant enhancement in release is likely due to free drug reversibly bound with the nanocarrier, resulting in slower apparent release from the dialysis tube and missed appearance in the receiver compartment after 1 h. After 6 h, the release of curcumin from both formulations was approximately 20%, followed by a plateau phase. After 7 days, loaded particles formulated using square Y-shaped and half-moon-shaped chips released 34.80 ± 2.95% and 32.72 ± 6.14% of the curcumin, respectively.

## 3. Discussion

### 3.1. Chip Manufacturing

In this work, four innovative micromixing patterns were developed. An FDM-based printing technique was used since it presents advantages such as the affordability of the materials and printers required and ease of initial use [35]. The latest photocuring 3DP technique, LCD printing, offers advantages such as affordable printers and good resolution [36]. Vat polymerisation allows for impressively small flow channel cross sections, but nevertheless wider channel dimensions were used in this work since it is often difficult to remove resin from the channels when their width is limited [37]. In addition, this study demonstrated that the developed microfluidic devices allow for the production of liposomes comparable in size to those produced using a commercial device. Therefore, 3DP is confirmed to be an effective technique for microfluidic device fabrication.

To the best of our knowledge, this is the first approach to liposome production using LCD-printed microfluidic devices. Microfluidic device fabrication using LCD printers has been previously described, although no studies in the literature report on these devices being applied to NP production [38]. Instead, FDM-printed microfluidic devices have been used for the synthesis of polymeric and lipidic NPs, but using different chip materials, channel patterns, and microfluidic parameters [35]. Nevertheless, 3D-printed NinjaFlex-based microfluidic devices have not previously been realized by FDM printing for liposome formulation.

### 3.2. Statistical Analysis: Comparison of Empty Liposomes

**Figure 9:**
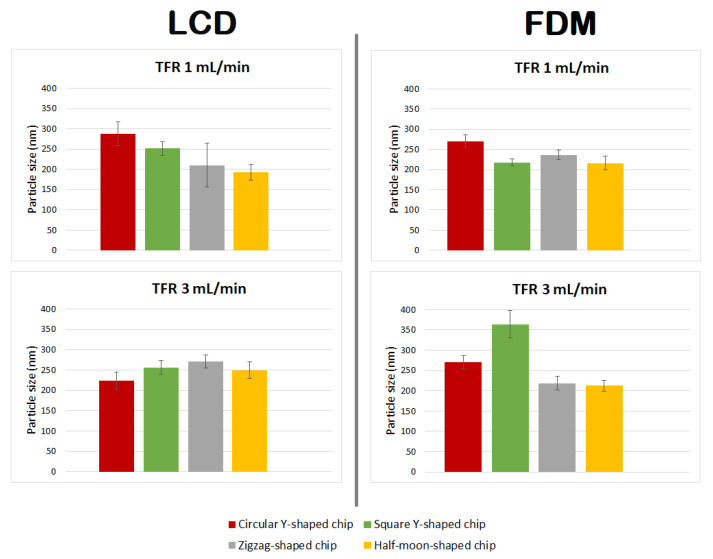
Comparison of particle size of DMPC liposome formulations prepared using circular Y-shaped, square Y-shaped, zigzag-shaped, and half-moon-shaped chips at TFRs of 1 and 3 mL min^−1^.

In this study, there was no distinct behaviour regarding particle sizing between liposomes manufactured with LCD- or FDM-printed chips, using different channel geometries, or due to different TFRs—even though the results show that these parameters have an influence upon particle characteristics (Figure 9). 

Liposomes manufactured using chips fabricated by LCD printer using TFR of 1 mL min^−1^, due to the relatively high standard deviation of the mean diameter of the liposomes formulated using the zigzag-shaped chip, showed a nonsignificant size difference with those formulated using square Y-shaped, half-moon-shaped, and commercial chips. Furthermore, there was a nonsignificant difference between particles achieved using half-moon-shaped and commercial chips at TFR of 1 mL min^−1^. Liposomes manufactured using circular Y-shaped, square Y-shaped, and half-moon-shaped chips showed a statistically significant difference in particle size between them. 

Regarding liposomes manufactured using chips fabricated by LCD printer using TFR of 3 mL min^−1^: NPs formulated using square the Y-shaped chip showed no statistically significant difference with those formulated using zigzag-shaped or half-moon-shaped chips; liposomes formulated using the other chips showed a statistically significant difference. Moreover, NPs formulated using the commercial chip showed no statistically significant difference with those formulated using circular Y-shaped, square Y-shaped, or half-moon-shaped chips at the same TFR.

Regarding liposomes manufactured using chips fabricated by FDM printer using TFR of 1 mL min^−1^: liposomes manufactured using the square Y-shaped chip showed no statistically significant difference with those formulated using the half-moon-shaped chip; liposomes formulated using the other chips showed a statistically significant difference between them and with those formulated with the commercial chip.

Regarding liposomes manufactured using chips fabricated by FDM printer using TFR of 3 mL min^−1^: NPs formulated using the zigzag-shaped chip showed no statistically significant difference with those formulated using the half-moon-shaped chip; NPs formulated with zigzag-shaped and half-moon-shaped chips showed a nonsignificant difference with those formulated with the commercial chip; liposomes formulated using the other chips showed a statistically significant difference.

The results showed a similar trend for mean particle size of liposomes manufactured using FDM printed chips at TFRs of 1 and 3 mL min^−1^, except for liposomes manufactured with the square Y-shaped chip. In this case, as the TFR increased from 1 to 3 mL min^−1^, an alteration in the particle size of liposomes manufactured using LCD chips was seen.

### 3.3. Formulation of Curcumin-Loaded Liposomes

**Figure 10:**
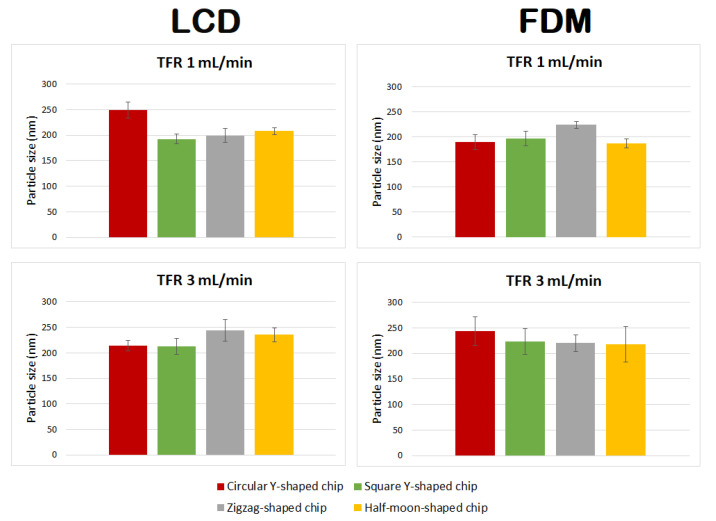
Comparison of particle size of curcumin-loaded liposomes prepared using circular Y-shaped, square Y-shaped, zigzag-shaped, and half-moon-shaped chips at TFRs of 1 and 3 mL min^−1^.

Curcumin-loaded liposomes achieved with different chip designs, chip manufacturing techniques, and TFRs differed in size from empty liposomes (Figure 10 and Table 3). In particular:

- Liposomes manufactured using chips fabricated by LCD printer using TFR of 1 mL min^−1^ showed no statistically significant difference between zigzag-shaped and square Y-shaped chips, or between zigzag-shaped and half-moon-shaped chips;
- Liposomes manufactured using chips fabricated by LCD printer using TFR of 3 mL min^−1^ showed no statistically significant difference between circular and square Y-shaped chips, or between zigzag-shaped and half-moon-shaped chips;
- Liposomes manufactured using chips fabricated by FDM printer using TFR of 1 mL min^−1^ using the zigzag-shaped chip are significantly different in size from those formulated with the other chips;
- Liposomes manufactured using chips fabricated by FDM printer using TFR of 3 mL min^−1^ showed no statistically significant difference between different chip designs.

Enhanced homogeneity in particle size was achieved in curcumin-loaded liposomes manufactured using different TFRs and microfluidic devices. Thus, in many cases, changing the combination of the parameters did not result in statistically different liposomes.

### 3.4. Encapsulation Efficiency and In Vitro Release

The encapsulation efficiency achieved for curcumin-loaded liposomes formulated using the microfluidic method was approximately 99.9% for the two formulations. Only one study was previously conducted regarding curcumin-loaded liposomes formulated by microfluidics, which provided lower loading capacity (~94%) using a commercial chip with different dimensions [27]. This work disclosed the first curcumin formulation with enhanced encapsulation efficiency; this was achieved using square Y-shaped and half-moon-shaped chips and different methods and experimental conditions than the study performed by Hamano et al. [27].

The release profile graph (Figure 8) shows a similar release profile for both formulations, with curcumin leakage after 48 h of 31.99% ± 3.20%, using the square Y-shaped chip, and of 28.33% ± 4.86%, using the half-moon-shaped chip. Thus, this alteration of the pattern of the chip channels did not significantly influence the release profile of the lipid or the drug in question; nevertheless, the square Y-shaped chip generated more homogeneously dispersed particles. Previous studies achieved a higher release (approximately 50% after 4 h) using a different release method and operative parameters [27].

## 4. Materials and Methods

### 4.1. Materials

**Figure 11:**
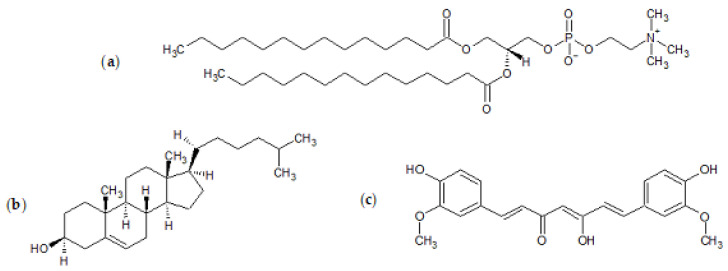
Chemical structures of (**a**) 1,2-dimyristoyl-sn-glycero-3-phosphocholine (DMPC), (**b**) cholesterol, and (**c**) curcumin.

The synthetic lipid (Figure 11) 1,2-dimyristoyl-sn-glycero-3-phosphocholine (DMPC), cholesterol, tablets of phosphate-buffered saline (PBS, pH 7.4), isopropyl alcohol, and ethanol ≥99.8% were all obtained from Sigma-Aldrich (Steinheim, Germany). Curcumin was acquired from TCI. NinjaFlex thermoplastic polyurethane (TPU) filament was obtained from iDig3Dprinting and Zortrax photopolymer resin white/ivory from Zortrax store.

### 4.2. Manufacturing of 3D-Printed Chips

Microfluidic devices were fabricated using Ultimaker 2+ FDM printer and UV LCD technology-based Zortrax Inkspire.

Ultimaker 2+ has a glass build plate with a 230 × 225 × 205 mm build space and a 0.8 mm nozzle diameter. The published literature on FDM printing polymers highlights the different mechanical properties of stiff and flexible polymers [39]. Stress-strain curves indicate that polymers such as PLA have a traditional deformation profile, while polymers such as NinjaFlex TPU filament exhibit an elastomeric behaviour [39]. The NinjaFlex filament was used in this study as a material for the printing since it is more flexible, and consequently less breakable, compared with polymers that follow the typical tensile curve. Prior to initiating device printing, the material was preheated, and an initial layer outline was created. Once the process starts, consecutive layers are deposited onto the preceding ones. The layer thickness depends on the quality required. 

Zortrax Inkspire uses a resin-based technology. The material used is the white/ivory photopolymer resin from the basic resin series developed to work with the Zortrax Inkspire. This resin is acrylate-based and solid layers are built, involving photopolymerization of acrylate monomers by the free radical mechanism. The UV LCD technology in Zortrax Inkspire allows for the curing of an entire layer of the model at once, and the device is printed layer by layer. Each layer is supported by either the platform, the previous layer, or support elements. Extra support elements and optimal orientation of the model and the supports can be adjusted with the Z-SUITE software in the processing stage. The STL model is then sliced into layers, and sent to the printer. After printing is complete, resin 3D prints require a postprocessing stage consisting of the removal of the support elements and the rinsing of the device with isopropyl alcohol in order to remove the liquid resin from the model.

### 4.3. Preparation of Liposomes by Microfluidics

Liposomes were prepared using a readymade microfluidic micromixer and 3D-printed microfluidic devices (Figure 4) connected to the Dolomite Microfluidic System. The commercial cartridge is 52 mm thick and 36 mm high with molded channels 300 μm in width and 130 μm in height with a staggered herringbone structure. To note, the cartridge channels for the commercial chip are smaller (1/3) with respect to the ones manufactured by 3DP. As previously mentioned in Section 2.2, phospholipids (DMPC) combined with cholesterol at a ratio of 2:1 were dissolved in ethanol at a concentration of 1 mg/mL. 

Fluids were delivered into the chip using two pressure pumps with respective fluidic connections in fluorinated ethylene propylene (PEF) of outside diameter (OD) 1/16 and inside diameter (ID) 250 μm. Flow rate ratio and TFR were controlled using two Mitos Flow Rate Sensors (0.2–5 mL min^−1^). The two chip inlets correspond to the lipid solution and the aqueous solution (PBS, pH 7.4). The liposome batch suspensions were collected from the outlet port, centrifuged at 14,800 rpm for 30 min in order to separate them from the supernatant, and rinsed three times with 1 mL of PBS in order to eliminate the organic solvent. 

### 4.4. Particle Sizing and ζ-Potential

Liposome size distribution (mean diameter and polydispersity index, PDI) were evaluated through dynamic light scattering (DLS) analyses performed on a NanoBrook Omni (Brookhaven Instruments, Holtsville, NY, USA). Prior to the analyses, each sample was diluted 1:100 with PBS pH 7.4. Formulations were then measured three times at 25 °C at a fixed angle of 90°. The ζ-potential was measured using the same instrument through phase analysis light scattering (PALS) studies.

### 4.5. Statistical Analysis: Comparison of Empty Liposomes

T-tests were performed in order to find evidence of significant differences in size between liposomes formulated with different chips; the results are shown in Table 4. The determination of a statistically significant difference between two means of size values is reported as a *p*-value [40]. Typically, if the *p*-value is below 0.05, the means are considered significantly different, and the lower the *p*-value, the greater the evidence that the two groups’ means are different [40].

### 4.6. Encapsulation Efficiency and In Vitro Release Studies

Encapsulation efficiency and release studies were conducted on liposomes formulated using square Y-shaped and half-moon-shaped chips fabricated by LCD printer. Dialysis tubes (dialysis tubing cellulose membrane, average flat width 10 mm, 0.4 in., MWCO 14000, Sigma-Aldrich) were sterilized in boiling water for 30 min and rinsed with deionized water before filling them with the liposome formulations [41]. Aliquots of 1 mL of each curcumin-loaded liposome suspension were placed into dialysis tubes and both ends were tied. Due to the very low solubility of curcumin in water (0.6 µg/mL in pure water), the release medium PBS pH 7.4 was supplemented with methanol (3%) and Tween 80 (0.5% *v*/*v*) [42]. Tubes were suspended in 7 mL of releasing medium and incubated at 37.0 ± 0.5 °C. After 1 h the release medium was removed, kept for the analysis of the percentage of unencapsulated curcumin, and replaced with fresh medium pre-equilibrated at 37.0 ± 0.5 °C. For release studies, a volume of 1 mL of the supernatant was collected after 30 min and 1, 2, 4, and 6 h, as well as after 5 and 7 days, and replaced with fresh pre-equilibrated medium. 

In order to determine the concentration of unencapsulated and released curcumin, samples collected were analysed for curcumin concentration using a UV–vis spectrophotometer (GENESYS 150, Thermo Scientific). Prior to analysis, a calibration curve of curcumin in ethanol was constructed at the maximum absorbance wavelength of curcumin (425 nm) [43].

The curcumin concentration at each point was calculated using Equation (1).

(1) $$\mathrm{Concentration}\;\left(\frac{\mu g}{\mathrm{mL}}\right)=\frac{\mathrm{Absorbance}-0.0045}{0.0179}$$

The encapsulation efficiency (%) was calculated using Equation (2).

(2) $$\mathrm{EE}\;\left(\%\right)=\frac{\mathrm{Ctot}-\mathrm{Cf}}{\mathrm{Ctot}}\times 100$$

where Ctot denotes the total concentration of curcumin in the dialysis tube at t0, on the basis of theoretical calculation, and Cf is the free drug concentration, given by the concentration of curcumin not encapsulated.

Release studies were conducted after 30 min and 1, 2, 4, and 6 h, as well as after 5 and 7 days. Given Ct as the concentration released at time t and at each time point, and Ce as the concentration of curcumin encapsulated, the percentage of cumulative release of curcumin is given by Equation (3).

(3) $$\mathrm{Cumulative}\;\mathrm{curcumin}\;\mathrm{release}\;\left(\%\right)=\frac{\mathrm{Ct}}{\mathrm{Ce}}\times 100$$

### 4.7. Statistical Analysis

When required, analysis of raw data via methods such as mean calculation or standard deviation was performed to simplify data for figures and tables. The raw data was obtained from three formulations of each type and each formulation was analysed three times respectively; thus, raw data was obtained by nine replicates throughout.

## 5. Conclusions

In this work, manufactured 3D-printed microfluidic devices were evaluated, and the developed chips resulted in effective production of lipid NPs. 

The microfluidic method was confirmed to be a simple and fast technique for liposome production. Empty and curcumin-loaded liposomes produced by microfluidics exhibited desirable size and a uniform size distribution profile.

The results demonstrated that TFR, chip manufacturing, material, and channel design have an influence on particle formulation. When designing a formulation study, it is of utmost importance to thoroughly investigate these parameters. In the perspective of scaling up the liposome manufacturing process using microfluidics, one future goal is to compare the quality and properties of microfluidic formulations in order to discover the correlation between the change in the operative parameters and the liposomes’ characteristics.

Moreover, there are limitations associated with the administration of liposomes above 200 nm, such as the removal of liposomes from the body by the mononuclear phagocyte system (MPS), limiting the targeting potential of NPs [44]. As for future perspectives, optimization of the process parameters may lead to optimization of the particles’ dimensions.

## Figures and Tables

**Table 1:** Mean particle size, polydispersity index (PDI), ζ-potential, and mobility of liposomes prepared using circular Y-shaped, square Y-shaped, zigzag-shaped, and half-moon-shaped chips (**a**) fabricated by LCD printer using TFR of 1 mL min^−1^, (**b**) fabricated by LCD printer using TFR of 3 mL min^−1^, (**c**) fabricated by FDM printer using TFR of 1 mL min^−1^, and (**d**) fabricated by FDM printer using TFR of 3 mL min^−1^.

	Chip Shape	Mean Diameter (nm)	PDI	ζ-Potential (mV)	Mobility (μmcm/Vs)
(**a**)	Circular Y	287.99 ± 29.72	0.259	−8.64 ± 2.63	−0.67 ± 0.20
	Square Y	251.14 ± 16.94	0.241	−5.93 ± 2.19	−0.46 ± 0.17
	Zigzag	210.46 ± 54.06	0.268	−10.11 ± 2.15	−0.79 ± 0.17
	Half–moon	193.42 ± 19.41	0.249	−9.81 ± 3.30	−0.77 ± 0.26
(**b**)	Circular Y	224.59 ± 20.93	0.271	−7.17 ± 2.34	−0.56 ± 0.18
	Square Y	256.52 ± 16.29	0.263	−6.49 ± 2.03	−0.51 ± 0.16
	Zigzag	271.21 ± 15.76	0.247	−8.22 ± 2.62	−0.64 ± 0.20
	Half–moon	250.00 ± 20.99	0.215	−6.53 ± 2.06	−0.51 ± 0.16
(**c**)	Circular Y	270.15 ± 15.48	0.255	−7.41 ± 2.14	−0.58 ± 0.17
	Square Y	217.39 ± 8.74	0.198	−7.55 ± 2.46	−0.59 ± 0.19
	Zigzag	236.43 ± 11.62	0.240	−7.57 ± 2.82	−0.59 ± 0.22
	Half–moon	216.17 ± 16.77	0.250	−6.21 ± 1.75	−0.48 ± 0.14
(**d**)	Circular Y	270.69 ± 16.29	0.277	−4.95 ± 2.00	−3.39 ± 0.15
	Square Y	364.05 ± 33.42	0.300	−5.18 ± 2.24	−0.40 ± 0.17
	Zigzag	218.94 ± 16.27	0.263	−6.85 ± 2.66	−0.54 ± 0.21
	Half–moon	212.65 ± 13.13	0.259	−5.54 ± 2.08	−0.43 ± 0.16

**Table 2:** Mean particle size, polydispersity index (PDI), ζ-potential, and mobility of curcumin-loaded liposomes prepared using circular Y-shaped, square Y-shaped, zigzag-shaped, and half-moon-shaped chips (**a**) fabricated by LCD printer using TFR of 1 mL min^−1^, (**b**) fabricated by LCD printer using TFR of 3 mL min^−1^, (**c**) fabricated by FDM printer using TFR of 1 mL min^−1^, and (**d**) fabricated by FDM printer using TFR of 3 mL min^−1^.

	Chip Shape	Mean Diameter (nm)	PDI	ζ-Potential (mV)	Mobility (µmcm/Vs)
(**a**)	Circular Y	249.18 ± 15.39	0.226	−9.83 ± 4.59	−0.77 ± 0.36
	Square Y	192.23 ± 9.54	0.211	−8.22 ± 9.93	−0.92 ± 0.31
	Zigzag	199.49 ± 13.13	0.189	−8.03 ± 2.36	−0.63 ± 0.18
	Half–moon	207.98 ± 7.14	0.183	−10.86 ± 2.23	−0.85 ± 0.17
(**b**)	Circular Y	214.32 ± 9.90	0.210	−9.68 ± 1.67	−0.76 ± 0.13
	Square Y	212.69 ± 15.48	0.166	−9.95 ± 3.15	−0.78 ± 0.25
	Zigzag	244.41 ± 21.03	0.199	−5.27 ± 2.13	−0.41 ± 0.17
	Half–moon	235.53 ± 13.30	0.168	−6.51 ± 3.74	−0.51 ± 0.29
(**c**)	Circular Y	189.81 ± 14.64	0.318	−10.63 ± 3.38	−0.83 ± 0.26
	Square Y	196.64 ± 14.72	0.176	−8.85 ± 2.62	−0.69 ± 0.20
	Zigzag	224.25 ± 7.03	0.124	−11.21 ± 2.77	−0.87 ± 0.22
	Half–moon	186.91 ± 9.45	0.195	−4.43 ± 2.20	−0.35 ± 0.17
(**d**)	Circular Y	243.77 ± 28.00	0.239	−13.07 ± 3.20	−1.02 ± 0.25
	Square Y	222.84 ± 25.26	0.152	−6.45 ± 3.21	−0.50 ± 0.25
	Zigzag	220.32 ± 16.26	0.186	−8.68 ± 2.69	−0.68 ± 0.21
	Half–moon	217.88 ± 35.10	0.225	−5.72 ± 2.29	−0.45 ± 0.18

**Table 3:** Values tabulated are two-tailed *p*-values calculated from mean sizes of curcumal liposomes formulated with the developed chips using LCD and FDM printers at TFRs of 1 and 3 mL min^−1^.

Chip Design	LCD		FDM	
	TFR 1 mL min^−1^	TFR 3 mL min^−1^	TFR 1 mL min^−1^	TFR 3 mL min^−1^
Circular Y-shaped–Square Y-shaped	0.003	0.794	0.339	0.116
Circular Y-shaped–Zigzag-shaped	0.012	0.002	0.029	0.049
Circular Y-shaped–Half-moon-shaped	0.009	0.002	0.626	0.104
Square Y-shaped–Zigzag-shaped	0.201	0.004	>0.000	0.805
Square Y-shaped–Half-moon-shaped	0.001	0.004	0.118	0.736
Zigzag-shaped–Half-moon-shaped	0.113	0.303	0.001	0.853

**Table 4:** Values tabulated are two-tailed *p*-values calculated from mean sizes of liposomes formulated with commercial and developed chips using LCD and FDM printers at TFRs of 1 and 3 mL min^−1^.

Chip Design	LCD		FDM	
	TFR 1 mL min^−1^	TFR 3 mL min^−1^	TFR 1 mL min^−1^	TFR 3 mL min^−1^
Circular Y-shaped–Square Y-shaped	0.007	0.003	0.008	0.021
Circular Y-shaped–Zigzag-shaped	0.003	0.058	<0.000	0.012
Circular Y-shaped–Half-moon-shaped	0.004	0.021	0.007	0.004
Square Y-shaped–Zigzag-shaped	0.058	0.070	0.001	0.003
Square Y-shaped–Half-moon-shaped	0.013	0.473	0.849	0.008
Zigzag-shaped–Half-moon-shaped	0.394	0.029	0.010	0.381
Commercial–Circular Y-shaped	0.004	0.662	0.002	0.019
Commercial–Square Y-shaped	0.012	0.106	0.002	0.003
Commercial–Zigzag-shaped	0.189	0.018	0.040	0.406
Commercial–Half-moon-shaped	0.320	0.234	0.004	0.211

## Data Availability

Data available on request due to restrictions.
